# No effect of unacylated ghrelin administration on subcutaneous PC3 xenograft growth or metabolic parameters in a *Rag1^-/-^* mouse model of metabolic dysfunction

**DOI:** 10.1371/journal.pone.0198495

**Published:** 2018-11-20

**Authors:** Michelle L. Maugham, Inge Seim, Patrick B. Thomas, Gabrielle J. Crisp, Esha T. Shah, Adrian C. Herington, Kristy A. Brown, Laura S. Gregory, Colleen C. Nelson, Penny L. Jeffery, Lisa K. Chopin

**Affiliations:** 1 Ghrelin Research Group, Translational Research Institute – Institute of Health and Biomedical Innovation, Queensland University of Technology, Brisbane, Queensland, Australia; 2 Australian Prostate Cancer Research Centre - Queensland, Translational Research Institute – Institute of Health and Biomedical Innovation, Queensland University of Technology, Brisbane, Queensland, Australia; 3 Comparative and Endocrine Biology Laboratory, Translational Research Institute – Institute of Health and Biomedical Innovation, Queensland University of Technology, Brisbane, Queensland, Australia; 4 Skeletal Biology and Forensic Anthropology Research Laboratory, School of Biomedical Sciences, Queensland University of Technology, Brisbane, Queensland, Australia; 5 Integrative Biology Laboratory, College of Life Sciences, Nanjing Normal University, Nanjing, China; 6 Department of Medicine, Weill Cornell Medicine, New York City, New York, United States of America; University of Cordoba, SPAIN

## Abstract

Ghrelin is a peptide hormone which, when acylated, regulates appetite, energy balance and a range of other biological processes. Ghrelin predominately circulates in its unacylated form (unacylated ghrelin; UAG). UAG has a number of functions independent of acylated ghrelin, including modulation of metabolic parameters and cancer progression. UAG has also been postulated to antagonise some of the metabolic effects of acyl-ghrelin, including its effects on glucose and insulin regulation. In this study, *Rag1*^*-/-*^ mice with high-fat diet-induced obesity and hyperinsulinaemia were subcutaneously implanted with PC3 prostate cancer xenografts to investigate the effect of UAG treatment on metabolic parameters and xenograft growth. Daily intraperitoneal injection of 100 μg/kg UAG had no effect on xenograft tumour growth in mice fed normal rodent chow or 23% high-fat diet. UAG significantly improved glucose tolerance in host *Rag1*^*-/-*^ mice on a high-fat diet, but did not significantly improve other metabolic parameters. We propose that UAG is not likely to be an effective treatment for prostate cancer, with or without associated metabolic syndrome.

## Introduction

The peptide hormone ghrelin is a circulating appetite-stimulating hormone which regulates a number of other biological processes [1–3]. These include metabolism and energy balance [1–4], and diseases such as cancer [5]. Ghrelin acts via its cognate receptor, the growth hormone secretagogue receptor 1a (GHSR1a), a G protein-coupled receptor [6], and one or more unknown alternative receptors [7–10]. In order to activate GHSR1a at physiological concentrations, ghrelin must be acylated at its third residue, a serine [11, 12], by the enzyme ghrelin *O*-acyl transferase (GOAT) [11, 12].

The major circulating form of ghrelin is its unmodified form, unacylated ghrelin (UAG; also known as DAG). UAG, which does not directly stimulate feeding [5], was initially considered to be functionally inactive, but is now appreciated to bind to and activate a distinct, unknown receptor [4, 13–18] and have a number of functions [19–22]. UAG plays roles in the regulation of glucose and energy balance and has effects on cell proliferation [19–23]. Importantly, it may oppose some of the effects of acyl-ghrelin [16, 24–26] by preventing the rise in circulating glucose and insulin associated with acyl-ghrelin administration in rodents [22, 26, 27]. From these studies, it is apparent that UAG is an endocrine hormone in its own right [20]. UAG and the truncated, cyclised UAG analogue AZP-531 prevented the development of pre-diabetes in C57BL/6 mice fed a high-fat diet for two weeks, highlighting a potential of unacylated forms of ghrelin as treatments for metabolic syndrome [27]. In human trials, UAG had similar effects, improving glycaemic control and insulin sensitivity in patients with type 2 diabetes mellitus [28] and improving glucose handling and reducing free fatty acids in healthy subjects when administered overnight as a continuous infusion [29]. AZP-531 also had beneficial effects on glucose balance and led to weight loss in patients with type 2 diabetes mellitus in a phase I clinical trial [30]. Similar benefits have been observed in patients with Prader-Willi syndrome, a genetic disorder associated with hyperghrelinaemia and obesity [31].

Close to two decades of work has firmly established a role for the ghrelin axis in cancer [5, 32–35]. This includes prostate cancer, a classical endocrine-related cancer and the most commonly diagnosed cancer in American men after skin cancer [36], where acyl-ghrelin increases cell proliferation and migration [5, 14, 37–46]. UAG also has functional effects in several cancers, including prostate cancer [5, 32–34, 43]. In the PC3 prostate cancer cell line UAG has a biphasic effect, reducing cell proliferation at supraphysiological levels (10nM-1μM) [14].

Studies investigating the role of UAG in prostate cancer have been limited to *in vitro* experiments. *In vivo* studies are required, however. Obesity, overweight, and co-morbidities (including hyperinsulinaemia) are now recognised as critical risk factors for numerous cancers [47–49]. These include cancer types with high-prevalence and mortality, such as tumours of the prostate, endometrium, breast, and gastrointestinal system [47–55]. Obesity and increased body mass have been associated with increased risk of advanced prostate cancer, more aggressive and high-grade disease, and increased risk of death from prostate cancer [56–59]. Castration-resistant prostate cancer (CRPC) occurs when prostate cancer recurs after remission from androgen-targeted therapies (ATT) [60]. Treatments for CRPC are limited and this stage of the disease often results in the formation of painful, metastatic bone lesions and associated morbidity and mortality [61–63]. Metabolic syndrome and hyperinsulinaemia are common side effects of ATT [64, 65] and may also further accelerate the progression to CRPC [48, 58, 66–68]. As UAG reduces prostate cancer proliferation *in vitro* [14] and has potential beneficial metabolic effects *in vivo*, we examined the effect of UAG in our model of metabolic dysfunction: *Rag1*^*-/-*^ mice fed a high-fat diet with subcutaneous prostate cancer cell line xenografts [69].

## Materials and methods

### Cell culture

Human prostate cancer cell lines were obtained from the American Type Culture Collection (ATCC, Manassas, VA, USA). The PC3 prostate cancer cell line was cultured in Roswell Park Memorial Institute 1640 medium (RPMI-1640) and supplemented with 10% (v/v) Fetal Calf serum (FCS) (Thermo Fisher Scientific, Waltham, MA, USA), 50 units/ml penicillin, and 100 μg/mL streptomycin (Thermo Fisher Scientific). Cells tested negative for *Mycoplasma*.

### Hyperinsulinaemic *Rag1*^*-/-*^ mouse model treated with unacylated ghrelin (UAG)

To determine the metabolic effect of UAG in an engraftable mouse model of hyperinsulinaemia [69], male recombination-activation gene deficient mice (B6.SVJ129-*Rag1*^*tm1Bal*^/Arc; *Rag1*^-/-^) (Jackson Laboratories, supplied by Animal Resource Centre, Murdoch, WA, Australia) were weaned onto a diet of normal chow (chow) or a Western-style, high-fat diet (HFD; 23% fat, SF04-027, Specialty Feeds, Glen Forrest, WA) [69]. After two weeks on the diet, mice were anaesthetised and subcutaneously injected into the left flank with 1×10^6^ PC3 cells diluted 1:1 in growth factor reduced, phenol red-free Matrigel (Corning, Corning, NY, USA). Tumours were allowed to grow until a volume of approximately 50–100 mm^3^ was reached, when mice were randomly divided into two experimental groups. Mice then received daily intraperitoneal injections of 100 μg/kg UAG (Mimotopes, Mulgrave, VIC, Australia) (*n* = 6 HFD, *n* = 10 chow), a dose previously determined to inhibit breast cancer growth *in vivo* [8], or phosphate buffered saline (PBS) control (*n* = 8 HFD, *n* = 10 chow) for 16 days. Tumour volume was calculated by measuring subcutaneous tumour length and width twice weekly using digital calipers (ProSciTech, Kirwan, QLD, Australia). Tumour volume was calculated using the equation ‘tumour volume = (width × length^2^)/2’ [70]. Body-weight was measured twice weekly.

In order to determine the metabolic effects of the diet, intraperitoneal (i.p.) glucose tolerance tests were performed (*n* = 7 chow PBS group; *n* = 6 chow UAG group; *n* = 5 chow PBS group; *n* = 6 chow PBS group) as previously described [69]. Briefly, mice were fasted for 16 hours and baseline glucose levels measured in tail-tip blood using OneTouch Ultra Blood Glucose Monitoring System test strips (Accu-Chek Performa, Roche, Basel, Switzerland). Glucose (20% solution, 2 g/kg) was injected i.p. and blood glucose levels assessed 15, 30, 60, and 120 minutes post injection. At experimental endpoint (fourteen days of treatment, or ethical endpoint) mice were euthanised using 70% carbon dioxide followed by cervical dislocation after death was confirmed. Ethical endpoint was based on the tumour volume reaching 1,000 mm^3^ or a combination of signs of stress including increased heart rate, inactivity, reduced interaction with cage mates, abnormal posture and/or >20% body weight loss. At endpoint, tumours and adipose tissue (epididymal fat pad and interscapular brown adipose tissue) were excised and weighed. Fasting blood glucose was measured at endpoint and blood collected post mortem by cardiac puncture for serum biochemical measurements.

All mice were housed under pathogen-free conditions in individually-ventilated cages, at a room temperature of 20–23 °C, with a 12-hour light-dark cycle. All methods were conducted in accordance with ethical guidelines and regulations and ethics approval from the University of Queensland and Queensland University of Technology Animal Ethics Committees and ethics approval for cell line (LNCaP) use was granted from the Queensland University of Technology Human Research Ethics Committee.

### Hormone measurement

Fasting serum insulin and total and acyl-ghrelin levels were determined by ELISA (EMD Merck Millipore Group, Darmstadt, Germany). Absorbance at 450 and 595 nm was determined using a FLUOstar Omega plate reader and software (BMG Labtech, Offenburg, Germany), with absorbance values interpolated using linear regression.

Surrogate indices of insulin resistance, insulin sensitivity, and steady state β-cell function were determined using the homeostatic model for assessment calculator (HOMA2), available from the Oxford Centre for Diabetes, Endocrinology and Metabolism [71], using measured fasting glucose and insulin levels. Unacylated ghrelin (UAG) levels were estimated by subtracting measured serum acyl-ghrelin levels from serum total ghrelin levels.

### Histological tissue analysis

Tissue was processed and embedded in paraffin before sectioning (5 μM sections). Immunohistochemistry was performed on tumour sections to investigate the expression of the proliferation marker Ki67 (rabbit anti-Ki67 primary antibody, undiluted, Roche, Basel, Switzerland) and CD31, a marker of angiogenesis (rabbit anti-mouse CD31 primary antibody diluted 1:50 in antibody diluent; Abcam, Cambridge, UK). Sections were counterstained with Mayer’s haematoxylin, dehydrated, and mounted with coverslips using D.P.X neutral mounting medium (Sigma-Aldrich) and observed using an Olympus BX41/702 microscope (U-CMAD3) (Shinjuku, Tokyo Metropolis, Japan).

### Statistics

Statistical analyses were performed using GraphPad Prism v.6.01 software (GraphPad Software, San Diego, CA). Kruskal-Wallis (three or more groups) and Mann-Whitney *U*-test (two groups) tests used for non-normally distributed data, while a two-way ANOVA with Tukey’s *post-hoc* test used for normally distributed data. *P* ≤ 0.05 was considered to be statistically significant.

## Results

### *Rag1*^*-/-*^ mice fed a high-fat diet show symptoms of metabolic dysregulation

**Fig 1:**
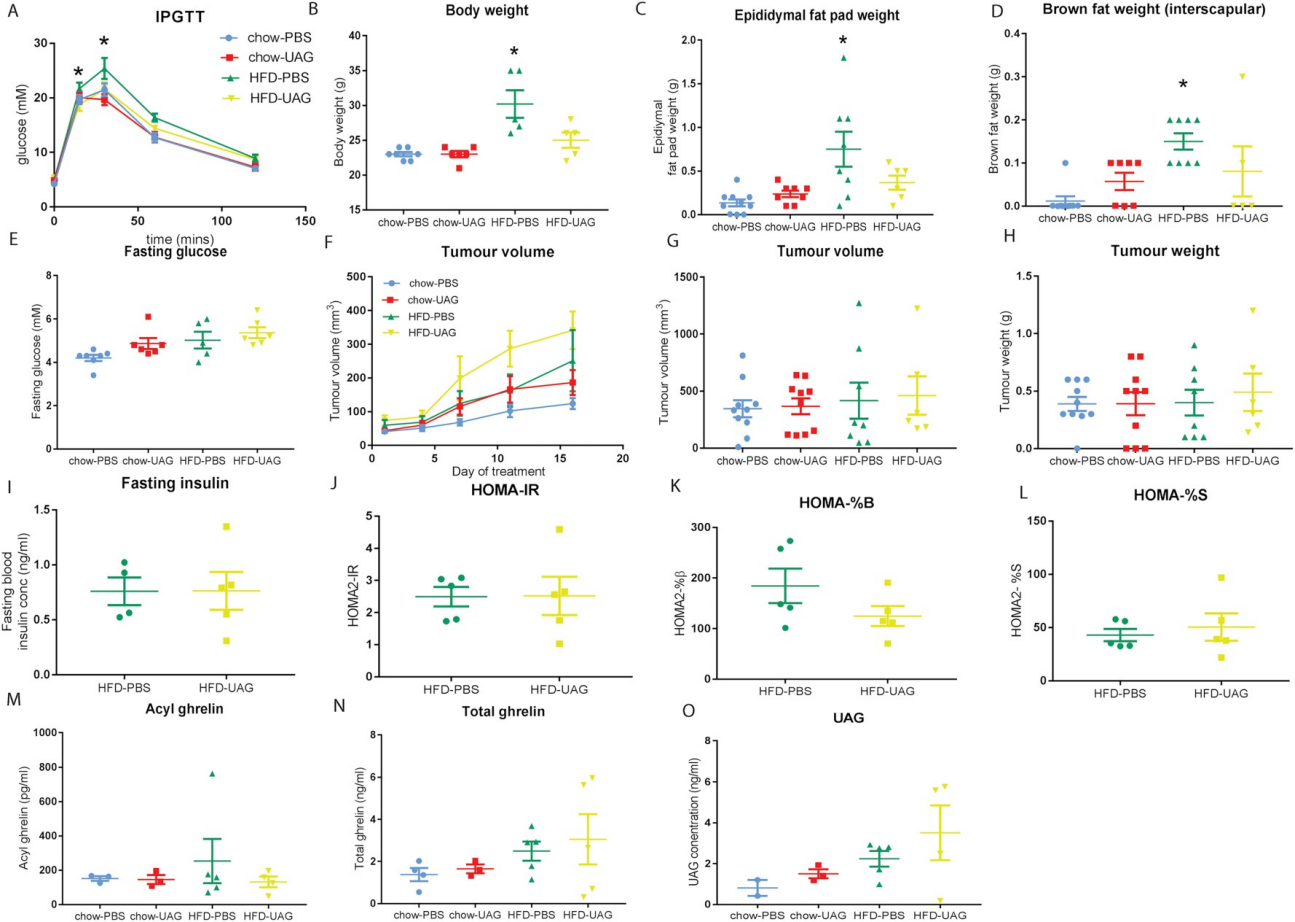
Unacylated ghrelin (UAG) affects glucose tolerance but has no effect on tumour volume or other metabolic parameters. *Rag1*^-/-^ mice fed a 23% high-fat diet (HFD) or chow were injected with subcutaneous PC3 xenografts and administered UAG (100μg/kg/day, i.p.) (*n* = 6 HFD, *n* = 10 LFD) or PBS control (*n* = 8 HFD, *n* = 10 chow) once tumours were palpable. Mean ± s.e.m. * *P* ≤ 0.05. (A) HFD-fed UAG-treated mice (*n* = 6) had significantly lower blood glucose 30 min post-glucose challenge compared to HFD-fed PBS treated mice (*n* = 8), determined by intraperitoneal glucose tolerance test (IPGTT). Mean ± s.e.m. Two-way ANOVA. * *P* = 0.025. (B) Body weight (g) of mice at endpoint was higher in the HFD-fed mice compared to the chow fed mice (*P<0.05), but was not different between the UAG and PBS groups (*P =* 0.08). (C) Epididymal fat pad weight (g) was greater in the HFD-PBS group compared to the chow fed-PBS group (*P<0.05), but not the UAG and PBS treatment groups (*P =* 0.26). (D) Interscapular brown adipose tissue weight (g) was increased in the HFD-PBS group compared to the normal chow groups (*P* = 0.0002), but not significantly different in UAG-treated mice compared to PBS-treated mice (*P* = 0.12). Mean ± s.e.m. Mann-Whitney *U*-test. (E) Fasting blood glucose (mM) was not altered in UAG-treated compared to PBS-treated mice on either diet (*P =* 0.50). Mean ± s.e.m. (F) Tumour volume (mm^3^) measured over time (*P =* 0.57), (G) and tumour volume (mm^3^) (*P =* 0.55) and (H) weight (g) at experimental endpoint were not significantly different between UAG- and PBS-treated mice fed HFD or chow. Mean ± s.e.m. Mann-Whitney *U*-test. (I) Fasting blood insulin (ng/ml) was not altered in UAG-treated compared to PBS-treated mice on either diet (*P =* 0.90). Mean ± s.e.m. Mann-Whitney *U-*test. (J) Insulin resistance (HOMA-IR) (*P =* 0.70), (K) steady state β-cell function (HOMA%B) (*P =* 0.22) and (L) insulin sensitivity (HOMA%S) (*P* = 0.70) were not altered in UAG-treated compared to PBS-treated mice or by either diet. (M) Plasma acyl-ghrelin was not altered in mice treated with UAG compared to other mice. (N) Plasma total ghrelin and (O) UAG levels in mice administered UAG (100μg/kg/day) compared to mice treated with PBS. Mean ± s.e.m. Mann-Whitney *U*-test.

As reported in our previous study [69], mice fed a Western-style 23% fat diet (HFD) developed symptoms of metabolic syndrome, including hyperinsulinaemia and increased lipid accumulation in the liver and skeletal muscle, compared to normal chow-fed controls. After 7 weeks on the diets, mice fed an HFD developed significantly impaired glucose tolerance 30 (*P =* 0.011, *n =* 7, Fig 1A) and 60 minutes (*P =* 0.019) after glucose challenge compared to control mice fed normal chow. Similarly, body-weight (*P =* 0.003, Fig 1B) and the weight of white adipose epididymal fat pad deposits (*n =* 8, *P =* 0.004, Fig 1C) and interscapular brown adipose tissue (*P =* 0.0002, *n =* 8, Fig 1D) were significantly greater at endpoint in HFD-fed mice compared to those fed normal chow. No significant differences in tumour volume or weight (Fig 1F–1H) were observed six weeks after subcutaneous xenograft implantation between mice fed HFD or normal chow. This was expected and mirrors previous results where significant differences in tumour size were observed only after eight weeks [69].

### No effect of intraperitoneal administration of UAG on PC3 xenograft growth in obese, hyperinsulinaemic *Rag1*^*-/-*^ mice

No significant differences in tumour volume over the treatment period or tumour weight (*P =* 0.57) and volume at endpoint (*P =* 0.55) were observed between UAG- and vehicle control (PBS)-treated obese mice (14 days of treatment) (Mann-Whitney test, Fig 1F–1H). Additionally, no difference in immunohistochemical staining of tumour xenografts for the proliferation marker Ki67 or the angiogenesis marker CD31 was observed between UAG or PBS treated groups, or mice fed normal chow or high-fat diet (HFD) (S1 Fig). Body-weight was reduced in the UAG treatment group fed HFD compared to the PBS treated group, however, the difference was not statistically significant (*P* = 0.08) (Fig 1B). No metabolic changes were observed in response to UAG treatment (Fig 1B–1E and 1I–1L). While there was a significant difference in fasting blood glucose 30 minutes following glucose challenge in HFD-fed UAG-treated mice compared to PBS controls at endpoint (after 16 days of treatment) (21.6 ± 1.2mM, *n* = 6 vs 25.4 ± 1.9mM, *n = 5*, *P =* 0.02, two-way ANOVA with *post-hoc* test, Fig 1A), this was not observed at other time points, suggesting that there was no major change in glucose tolerance. There was no significant difference in fasting blood glucose (*P* = 0.50), blood insulin concentration (*P* = 0.90, Mann-Whitney *U*-test, Fig 1I), insulin resistance (*P* = 0.70, Mann-Whitney *U-*test, Fig 1J), or insulin sensitivity (*P = 0*.*70*, Mann-Whitney *U-*test, Fig 1L) with UAG treatment compared to PBS control. Plasma acyl-ghrelin (Fig 1M) and total ghrelin levels (Fig 1N) were not altered by diet or UAG treatment, however increased serum UAG levels upon UAG administration was confirmed by ELISA (Fig 1O).

## Discussion

It has recently been recognised that UAG can act as a ghrelin inhibitor under some conditions, reducing ghrelin-mediated increases in plasma glucose [22, 26, 28, 72] and lipids [27, 29]. As the ghrelin axis also plays a role in the progression of a number of endocrine-related cancers [5, 32–34], including prostate cancer [5, 43], we hypothesised that UAG has beneficial effects in advanced prostate cancer associated with metabolic syndrome. To evaluate this hypothesis we examined the effect of UAG on prostate cancer cell line xenograft growth *in vivo*.

In our diet-induced hyperinsulinaemic *Rag1*^*-/-*^ mouse model [69], we investigated the effect of supraphysiological systemic UAG treatment (100μg/kg/day) on metabolic parameters and PC3 prostate cancer xenograft growth. No differences in metabolic parameters (fasting blood glucose, fasting blood insulin, insulin resistance, steady-state β-cell function, and insulin sensitivity) were observed following UAG treatment in HFD-fed mice. Other studies have found that UAG prevents insulin resistance and hyperglycaemia in short-term HFD-fed mice [73], observations which may stem from the ability of UAG to cross the blood-brain barrier and oppose the central actions of ghrelin on energy homeostasis [74]. Furthermore, in human clinical trials UAG improved glucose and lipid metabolism in healthy [29] and diabetic patients [28]. In our study, a decrease in body-weight, epididymal fat pad weight, and interscapular brown adipose tissue was observed in HFD-fed UAG-treated mice but this difference was not statistically significant. UAG did significantly reduce blood glucose levels at 30 minutes post-glucose challenge in HFD, but not mice on a normal chow diet, however. This is similar to other studies, which only found positive effects of UAG on glucose tolerance in obese patients [72]. Similarly, in clinical trials AZP-531 (a cyclised, truncated analogue of UAG) improved food-related behaviour, waist circumference, and glucose tolerance in Prader-Willi syndrome patients, but had no effect on body weight [31]. AZP-531 also prevents HFD-induced weight gain, insulin resistance, and impairment of glucose tolerance in mice [27].

To the best of our knowledge, this is the first report on the effects of UAG on cancer cell line xenograft growth *in vivo*. While our study and others show somewhat promising effects of UAG treatment on metabolic parameters, systemic UAG administration had no effect on prostate tumour xenograft size in mice fed a normal chow or high-fat diet. While preliminary, our study suggests that UAG administration, or targeting of endocrine UAG, may have limited therapeutic potential for prostate cancer–in patients with and without symptoms of metabolic syndrome.

## Supporting information

S1 Fig Unacylated ghrelin (UAG) has no effect on tumour histopathology or immunohistochemical markers for proliferation or angiogenesis. Immunohistochemistry for (A) the proliferation marker Ki67 and (B) the endothelial cell marker CD31, show no difference in positive staining (brown) in PC3 tumour xenografts from mice treated with UAG or PBS in the normal chow or high fat diet (HFD). Arrows show examples of positively stained cells.
(TIFF)
