# Supplementary figures and images for: No effect of unacylated ghrelin administration on subcutaneous PC3 xenograft growth or metabolic parameters in a Rag1-/- mouse model of metabolic dysfunction
Source: PLoS One. 2018 Nov 20;13(11):e0198495. doi: 10.1371/journal.pone.0198495 (PMC6245673; doi:10.1371/journal.pone.0198495)

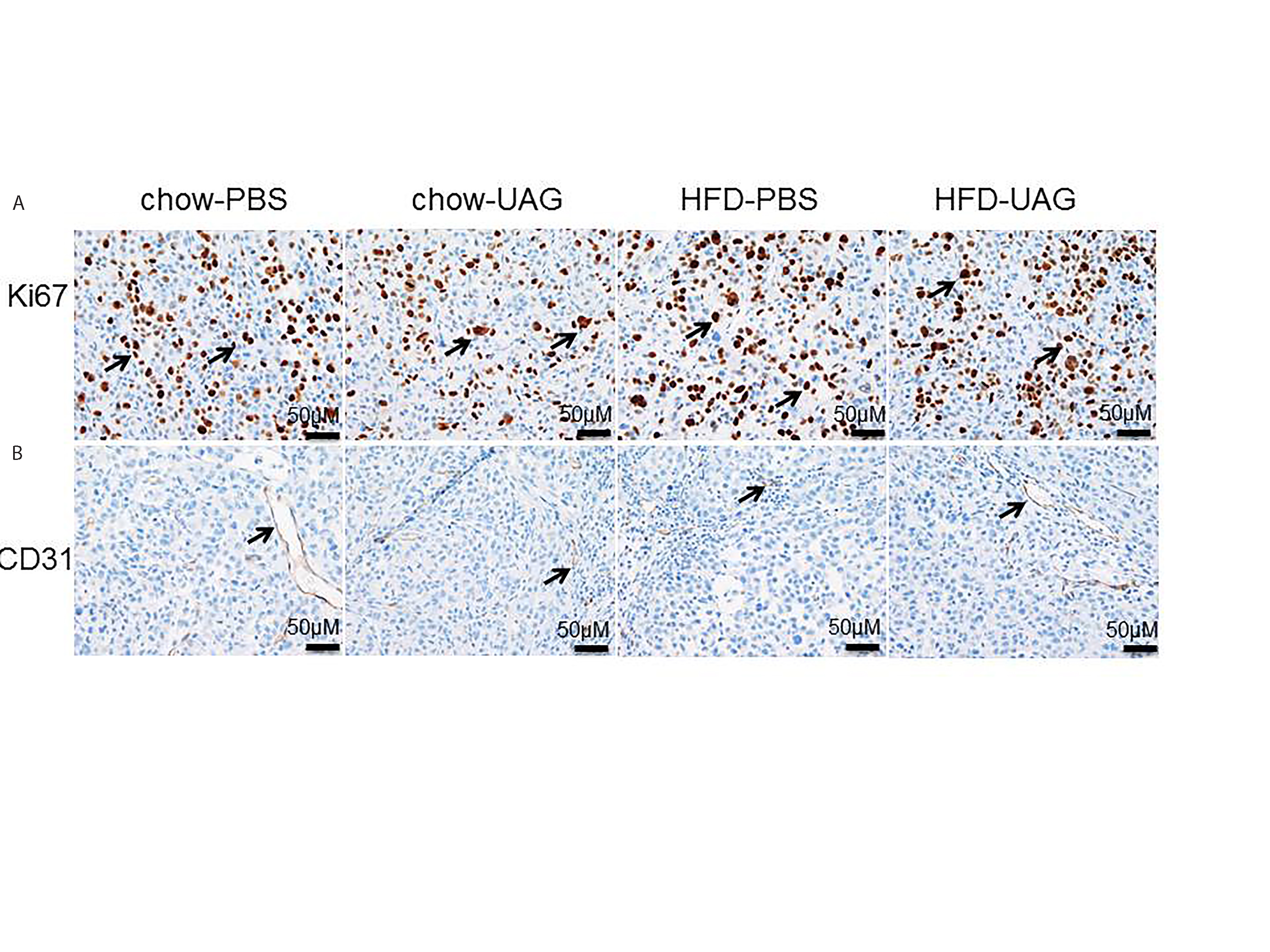

Supplement: S1 Fig — Immunohistochemistry for (A) the proliferation marker Ki67 and (B) the endothelial cell marker CD31, show no difference in positive staining (brown) in PC3 tumour xenografts from mice treated with UAG or PBS in the normal chow or high fat diet (HFD). Arrows show examples of positively stained cells. (TIFF) [file pone.0198495.s001.tiff]
